# Extreme Weather Events and Spiraling Debt: A Double Whammy for Bangladeshis Affected by Climate Change

**DOI:** 10.3389/fpsyg.2022.879219

**Published:** 2022-09-20

**Authors:** Shah Md Atiqul Haq

**Affiliations:** Department of Sociology, Shahjalal University of Science and Technology, Sylhet, Bangladesh

**Keywords:** climate change, coping strategies, double whammy, extreme weather events, routine experiences, spiraling debt

## Abstract

This study explores how people living in different areas of Bangladesh prone to extreme weather events (EWEs) in the form of floods, cyclones, or droughts perceive climate change, the impacts they suffer in the face of EWEs, and how they cope with their consequences. Qualitative data was collected through in-depth interviews with 73 respondents from three different areas of Bangladesh and subsequently analyzed. The results show that there are similarities and differences between respondents from regions with different vulnerabilities in terms of their views and perceptions about what climate change is its causes, the consequences of EWEs, and the strategies they adopt to cope with their effects. Respondents understood climate change based on their own local experiences of climate change and EWEs. A main finding is that people in all three areas are driven to borrow money in the face of these events as a survival strategy and to be able to continue to support their families. As the climate is set to change rapidly and EWEs to occur more frequently and regularly, it will become routine for those most vulnerable to them to have to cope and live with their impacts. Increased reliance on borrowing risks leading to a debt spiral for already vulnerable people. They are thus subject to a “double whammy”: on the one hand the direct effects of climate change and EWEs on their lives and livelihoods and on the other getting caught in a debt spiral sparked by times of crisis.

## Introduction and Background

Climate change and extreme weather events (EWEs)—floods, droughts, cyclones, changing precipitation patterns, and temperature variability (Adger et al., 2005)—can have a major effect on lives and livelihoods. Those affected are exposed to various negative impacts (Sultana, 2013), challenges, and losses and damage, economic and otherwise (Warner, 2010; Bhowmik et al., 2021). Ellis (2000) noted that the effects of major environmental change on livelihoods have human, natural, financial, social, and physical dimensions, all of which must be taken into account to understand how people are affected (e.g., income decline, deteriorating well-being, and food insecurity) by climate-related EWEs.

People living in areas particularly vulnerable to climate change are highly likely to experience various negative impacts and observe changes in their local environment and climatic conditions (Salick et al., 2009; Turner et al., 2009). These experiences in their local area, as well as their particular socio-economic, demographic, and cultural background, influence their beliefs and perceptions about climate change (Brouwer et al., 2007; Battaglini et al., 2009; Bunce et al., 2010; Speranza et al., 2010; Dunlap and Jacques, 2013; Fabiyi and Oloukoi, 2013; Haq and Ahmed, 2017). People who experience the negative impacts of local climate change, such as high precipitation, high temperatures, rising sea levels, and storm surges (Kleinosky et al., 2007; Younger et al., 2008; Poortiga et al., 2019), see climate change as a risk. Their level of concern depends on where they live and whether they experience certain types of EWE (Ayanlade et al., 2017). Perceptions are also shaped by individual attitudes and beliefs (Carlton and Jacobson, 2013).

Bangladesh is one of the world’s countries most vulnerable to climate change and EWEs and experiences various environmental and climate-change-related problems, including flooding, land degradation, soil erosion, and deforestation (Habiba et al., 2012; Haq, 2018; Haq et al., 2021). Floods, especially flash floods, are most common in the Haor region in the Northeast (Anik and Khan, 2012), while in Chattogram, cyclones have caused significant damage to property and infrastructure (Islam et al., 2018). People living in flood- and cyclone-prone areas face food shortages, insufficient drinking water, crop failure, unemployment, and loss of housing (Mallick and Vogt, 2014; Alam et al., 2017; Bhowmik et al., 2021). Cyclones, excessive rainfall and flooding, and salinity are the main climate risks to the livelihoods of coastal populations in Bangladesh (Aryal et al., 2020).

People spontaneously respond to critical circumstances and livelihood crises through various coping strategies (Burton, 2006; Detraz and Betsill, 2009; Sultana and Rayhan, 2012). Coping strategies depend on the type, severity, and nature of the EWEs (Haq, 2019; Bhowmik et al., 2021) and occur at the local level (Sultana and Rayhan, 2012; Haq et al., 2021). Vulnerable people consider short-term coping strategies, such as borrowing money or food, selling valuables, and hard work (Regassa and Stoecker, 2011; Tumaini and Msuya, 2017), and long-term ones, such as seasonal migration, changing their occupation, saving for emergencies, and changing their agricultural practices (Akhter et al., 2013; Ahmed and Haq, 2019; Bhowmik et al., 2021). Bhowmik et al. (2021) found that access to information and technology for populations vulnerable to climate change contributed to improved livelihoods through adaptation strategies such as changing agricultural practices and diversifying livelihood options.

Aryal et al. (2021) conducted a study involving farmers from East African (Ethiopia and Kenya) and South Asian countries (Bangladesh, India, and Nepal) exposed to different climate risks. Their findings for Bangladesh were distinct because its main climate risks are cyclones, salinity, flooding, and extreme rainfall, whereas for the other countries it is drought, followed by floods and extreme rainfall. The most common coping strategies were changing agricultural practices for farmers in Nepal and Kenya and saving and borrowing Bangladesh, India, and Ethiopia. In this study, many farmers in Bangladesh were receiving assistance from the government and NGOs to cope with the impacts of climate change. This specificity for Bangladesh prompted me to take the country as a case study focusing on three different EWE-prone locations within the country.

Studies have been conducted on specific areas affected by EWEs in Bangladesh exploring different coping strategies in areas affected by floods, cyclones, and droughts. For example, Bhowmik et al. (2021) studied how rural people in Bangladesh experience loss and damage from river flooding, flash flooding, and cyclones, and sought to capture their perceptions on the various EWEs that affect their lives and livelihoods. However, there have been few comparative studies examining how people in areas subject to different EWEs perceive and interpret climate change, how EWEs affect them, and how they respond to challenges. This study sets out to explore these issues in the case of Bangladesh. A better understanding of these issues can help the country’s policymakers rethink or update their climate change policies and reduce the vulnerability of people living in areas at high risk of EWEs (Aryal et al., 2020). These issues are important as the impacts of climate change and EWEs are unavoidable, and their increasing frequency and intensity worsen livelihood options, vulnerabilities, and the degree of losses and damage not only for Bangladeshis but also for populations in other developing countries (Bhowmik et al., 2021).

## Conceptual Framework

### Climate Change and Extreme Weather Events

To understand climate, climatologists analyze monthly and annual averages for weather-related parameters. Easterling et al. (2000) refer to extremely low or high daily temperatures and high daily or monthly precipitation events as extreme events. Whether a weather event is perceived as extreme depends on the context (Zwiers et al., 2011). Increased climatic variability is manifested in more frequent and unpredictable weather extremes or “weather shocks.” These are likely to make poor households even more vulnerable (Skoufias, 2012; Winsemius et al., 2015; Hallegatte et al., 2016) and may exacerbate the incidence, severity, and persistence of poverty in developing countries such as Bangladesh.

Bangladesh’s geographic features mean that it is vulnerable to EWEs (Karim and Mimura, 2008). The country has seen changes in rainfall and temperature patterns as well as severe EWEs (Shahid, 2012), including 191 floods, cyclones, or droughts between 1999 and 2018 (Eckstein et al., 2019), and most recently the devastating flooding in the northeastern part of the country. Increasing EWEs in the country imply greater vulnerability (Toufique and Islam, 2014) and have translated into significant loss of life and property and damage to people living in vulnerable areas (Ayeb-Karlsson et al., 2016).

### Perceptions About Climate Change

Perceptions and beliefs regarding climate change vary across communities and regions around the world (Dunlap and Jacques, 2013; Fabiyi and Oloukoi, 2013). Individual beliefs and attitudes toward climate change is built on knowledge shaped by cultural and ecological contexts and social values and influenced by socio-demographic factors (Turner et al., 2009; Haq and Ahmed, 2017, 2020; Adzawla et al., 2019; Ahmed and Haq, 2019; Dias et al., 2020). Some may feel climate change is caused by human activities (Dunlap and Jacques, 2013; Shukla et al., 2016) and others that it God’s response to human sin (Debela et al., 2015; Abegunde, 2017; Haq and Ahmed, 2017).

In Bangladesh, studies show that factors such as experiences of EWEs in one’s own locality (Haq and Ahmed, 2019; Chowdhury et al., 2021; Ahmed et al., 2022), age, gender, education, religion, marital status, occupation, income, size of landholdings, and exposure to mass media are correlated with and explain variations in perceptions about climate change (Anik and Khan, 2012; Huda, 2013; Haq and Ahmed, 2017). However, not all factors are equally influential or statistically significant, and their influence differs from one study area to another (Haq and Ahmed, 2017; Ahmed and Haq, 2019).

### Strategies for Coping With Climate Change Impacts

People’s livelihoods are a major factor in their vulnerability to current problematic conditions and future change, and an important part of documenting vulnerability is to understand the ways in which livelihoods are exposed to and respond to changing conditions (Sultana et al., 2020). Frequent EWEs, particularly floods, affect the livelihoods of millions of people in Bangladesh (Ahmed et al., 2019b; Islam et al., 2022). The most immediate impact of floods, cyclones, and droughts is damage to standing crops, which primarily affects smallholder farmers (Burton, 2006; Azad et al., 2013; Haq, 2018). Climate change brings households into poverty due to the loss of assets, crops, and income as well as food price shocks (Skoufias, 2012; Hallegatte et al., 2016). Poor people are invariably more vulnerable to climate-related shocks as they have fewer resources to draw on and receive less support from family, the community, the financial system, and even social safety nets to cope with and overcome the challenges (Hallegatte et al., 2016; Islam et al., 2022). These shocks also affect economic growth and increase poverty among poor vulnerable people, which can make it harder for a country to eradicate poverty (Skoufias, 2012; Hallegatte et al., 2016). In addition, shocks from EWEs result in increases in gender violence and sexual harassment (Islam et al., 2022). For instance, during EWEs, people go to shelter house and many affected people stay there and young women face the problem in Bangladesh (Ahmed et al., 2019b). As people’s income depends on agriculture, when there is crop loss due to such shocks, then small holder farmers feel pressure for managing their livelihoods as they are in financial crisis and that result in increased the domestic violence (Choudhury and Haque, 2016; Caridade et al., 2022).

Coping strategies are a spontaneous response of livelihood systems to a crisis, primarily at the local level (Aryal et al., 2021). For example, Snel and Staring (2001) mention that individuals and households in poor socioeconomic situations cut back on spending or find ways to earn extra income to be able to pay for basic needs in the face of EWEs. People first turn to coping mechanisms with short-term effects, such as dipping into savings or selling off assets, and when these are insufficient to meet needs, they may turn to longer-term strategies, such as withdrawing children from school (Cameron, 2001). In extreme cases, when few people in the community have surplus food, food-insecure households are forced to go to local grocers and ask for food on credit (Dafermos et al., 2018). In the absence of assets that can be used as collateral, as required by formal lending institutions, poor and marginalized households rely on informal sources of credit with high interest rates to be repaid through casual seasonal labor, which is likely to exacerbate food insecurity (Wisner et al., 2004; Regassa and Stoecker, 2011; Shikuku et al., 2017).

Aryal et al. (2021), in their examination of the main climate risks faced and adaptation strategies used by farmers in Ethiopia, Kenya, Bangladesh, India, and Nepal, found that cyclones and salinity are specific to Bangladesh while drought is prevalent in the other countries. Farmers’ responses to climate change included changing their farming practices, sustainable land management, reducing consumption, selling off assets, using savings and borrowing, and seeking alternative employment and assistance from governments or NGOs. Ahmed and Haq (2019) found that the Khasia and Tripura, two of Bangladesh’s indigenous groups, use traditional knowledge to mitigate the effects of climate change and engage in crop diversification, changing plantation and harvesting practices, and using short-season crops. Other local examples of behavioral change and adaptive strategies by vulnerable families include duck rearing, floating gardens, wave protection walls, cage aquaculture, canal re-excavation, and dam construction (Anik and Khan, 2012), and income diversification, especially for wealthier families (Brouwer et al., 2007). Ahmed et al. (2019a) found the main coping strategies for indigenous people in Naogaon (northwest Bangladesh) during droughts include water use reduction due to having to haul water long distances.

There is also a gender dimension in relation to coping and adaptation strategies: for example, Aryal et al. (2021), in their study in East Africa and South Asia, found significant differences between male- and female-headed households in their choice of strategies, with male-headed households more likely to seek additional work and change agricultural practices and households headed by women or older people more likely to reduce consumption and rely on savings and loans.

## Methodology

### Research Design and Study Location

Yin (2003) recommends a case study design that aims to answer “why” and “how” questions and relate the phenomena under study to contextual conditions. Accordingly, this study explores *how* experiences of the negative impacts of climate change for people living in areas vulnerable to EWEs influence what they think climate change is and leads them to consider different coping strategies for survival and livelihood, and *why* there are differences in coping strategies against different types of EWEs. Bangladesh, one of the most densely populated countries in the world, experiences flooding every year and is also prone, as previously noted, to cyclones and drought, and sea level rise poses a severe threat to the country. Bangladesh is thus a good setting for comparative study of perceptions about climate change, the impacts of various EWEs, and people’s coping strategies for EWEs. Creswell (2014) stated that research into the bases of human action in any given situation should focus on time and space. Also important is how people define climate change according to the contextual conditions in which they live (Miles and Huberman, 1994; Baxter and Jack, 2008).

**Figure 1:**
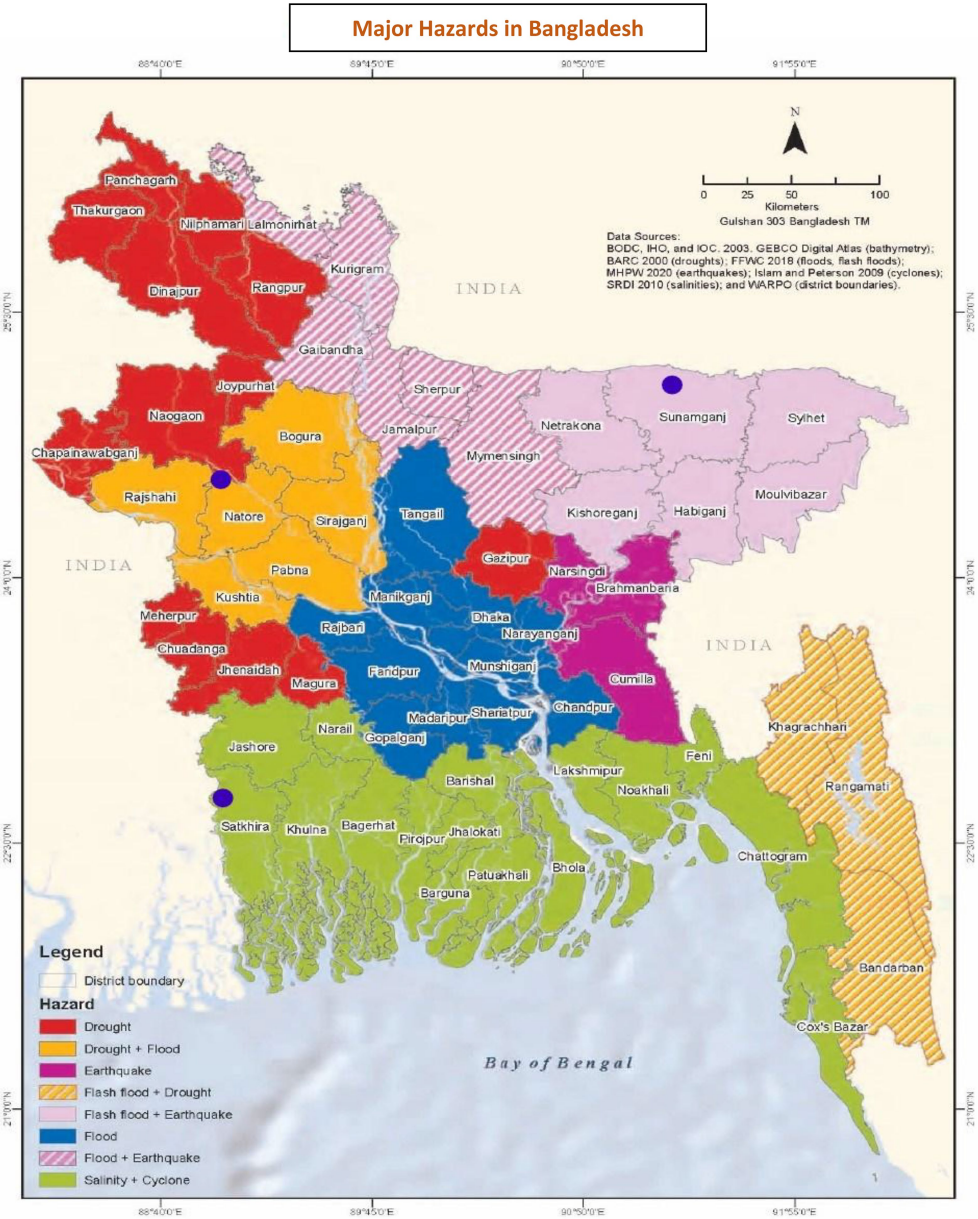
Areas vulnerable to different types of extreme weather event (EWE) and study locations (Bangladesh). The purple dots indicate the locations of the three upazilas included in this study. Source: https://www.adb.org/sites/default/files/publication/760781/bangladesh-climate-disaster-risk-atlas-volume2-cover-pgxxiv.pdf.

This study uses purposive sampling (Russell et al., 2005) to select upazilas (an administrative region functioning as a sub-unit of a district of Bangladesh). Tahirpur Upazila in Sunamganj District (in the Northeast) was purposively selected as a flood-prone area, Shyamnagar Upazila in Satkhira District (in Chittagong, in the southwest) as a cyclone-prone area, and Lalpur Upazila in Natore District (in the northwest) as a drought-prone area. Tahirpur Upazila, a frequently studied wetland, is prone to flash floods (Haq and Ahmed, 2017; Haq, 2018; Kamruzzaman and Shaw, 2018; Ahmed et al., 2019b) and is the most flood-affected area in Sunamganj (Kamal et al., 2018; Bhowmik et al., 2021). Shyamnagar Upazila was severely affected by Cyclone Aila in 2009 (Mallick et al., 2017). Lalpur Upazila is the most climate sensitive of the three and has experienced severe droughts (Eyasmin et al., 2021). Figure 1 shows the locations of the study.

### Sampling and Data Collection

Convenience sampling, a non-probability sampling technique, was used to collect qualitative data through in-depth interviews. Sampling considered factors such as the availability of respondents for an interview, their accessibility, and their interest in participating in the study. The target groups were married men 30–34 years old and married women 20–24 years old. People in these age groups were chosen because they have past and present experiences with the impacts of climate change and these may influence the coping strategies they choose given their long remaining lifespan into a future with frequent EWEs and changes in climatic conditions. Married people were chosen for their presumed sense of responsibility in terms of maintaining their livelihood and securing their children’s future. Responsibility as family breadwinners and potential longevity were thus the main criteria leading us to select these groups. Interviews were conducted individually, and in order to ensure that participants were not influenced by their spouse, only one spouse in any couple was interviewed, not both.

The information collected on what people think climate change means and why it happens, their experiences with the effects of EWEs, and their coping strategies against their negative effects was designed to yield insight into how people early in their married life perceive the inevitable climate-change crisis (Malak et al., 2020). The qualitative questionnaire was administered with the help of eight research assistants (four men and four women) who were studying social sciences at Shahjalal University of Technology, Sylhet. The research assistants were knowledgeable in research methods, were competent in survey administration, and had received training in data collection procedures before the interviews were conducted. Each participant was informed of the purpose of the study and was told that the information they provided would remain confidential, would not be disclosed, and would be used only for the purposes of this study. If they agreed to participate, they then provided written informed consent. The researcher developed the data collection tools. These included a semi-structured questionnaire on socio-demographic characteristics (gender, religion, age, and education level) and an interview guide listing a set of questions or topics to be raised and explored during the interview (e.g., How do you define climate change? What impacts do you face from EWEs?, and How do the address and overcome the challenges?).

For the flood-prone area (Tahirpur Upazila), we met with locally elected members of the union parishad (the lowest level of local government) as key figures to learn about the study site and explain the research objectives. They shared that there were 180 residents living in the two villages, Chiksa and Jamalgar, including people in our target groups. We visited each household to obtain the names and contact details of the married men aged 30–34 and the married women aged 20–24 in the villages. Using the lists, we collected information using convenience sampling in the form of guideline questions for the in-depth interviews and discussed with each respondent the possibility of an in-depth interview, following up by telephone to schedule it. Before each interview we informed participants of the research objectives, assured the confidentiality of the information they would give us, and obtained their consent to be recorded. We interviewed at total of 27 people in Tahirpur Upazila (eight men and 19 women). The relative number of male respondents is small because they tended to work until late in the evening before returning home. The average interview time for this area was 52 min (see Table 1).

**Table 1:** Socio-demographic characteristics of respondents.

**Characteristic**		**Tahirpur Upazila, Sunamganj (Flood-prone)**	**Shyamnagar Upazila, Satkhira (Cyclone-prone)**	**Lalpur Upazila, Natore (Drought-prone)**	**Total**
Gender	Male	30% (8)	54% (13)	36% (8)	40% (29)
	Female	70% (19)	46% (11)	64% (14)	60% (44)
	Total	100% (27)	100% (24)	100% (22)	100% (73)
Religion	Muslim	78% (21)	96% (23)	95% (21)	89% (65)
	Hindu	22% (6)	4% (1)	5% (1)	11% (8)
	Total	100% (27)	100% (24)	100% (22)	100% (73)
Mean age (years)	Male	31	31	32	31
	Female	22	22	23	22
	Total	25	27	27	26
Education	No schooling	30% (8)	4% (1)	5% (1)	14% (10)
	Primary	56% (15)	38% (9)	9% (2)	36% (26)
	Secondary	10% (3)	33% (8)	55% (12)	32% (23)
	Higher secondary	0	21% (5)	5% (1)	8% (6)
	Honors/degree	4% (1)	0	13% (3)	5% (4)
	Masters or higher	0	4% (1)	13% (3)	5% (4)
	Total	100% (27)	100% (24)	100% (22)	100% (73)
Interview duration	Total minutes	1,395	1,070	1,170	3,635
	Mean (minutes)	52	45	53	50

In the cyclone-prone area (Shyamnagar Upazila), the same research assistants met with members of the union parishad there to find out which were the most affected villages in the area and then collected household information about married men and women in the villages of Gabura and Khalishabunia. They found a total of 112 inhabitants in the target groups (57 from Gabura and 55 from Khalishabunia). They then prepared a list of the names and contact numbers of potential respondents. Finally, they conducted in-depth interviews with 24 respondents (11 men and 13 women).

The respondents in this upazila were eager to talk with us as they tended to confuse us with representatives from a donor agency. It was very difficult to talk with them about topics other than the cyclone. They hoped that if they described their difficulties from Cyclone Aila they would get more support from government and NGOs, saying that they had told many people about their suffering but had not received support, and requesting we add their name to a list for relief. In addition, it was challenging to talk with the young women, even if the interviewer was female: many left the interview without saying anything, and the female assistants had to pause recording many times. It was also difficult to obtain male respondents since they had to go to nearby areas or cities for work. Thus, the average interview time for this area was 45 min (see Table 1).

In the drought-prone area (Lalpur Upazila), a similar strategy was followed. The villages of Palideha and Gouripur were identified for interviews. The interviewers found 100 potential respondents in those villages in the target groups. In Gouripur, we had a problem recruiting men because most were day laborers and too tired for a 1-h interview in the evening. Therefore, the relative proportion of women respondents is high. People also seemed irritated by the hot weather, also inhibiting their willingness to be interviewed (Mohsenipour et al., 2018). In the end, we interviewed 22 people (eight men and 14 women), and the average duration of each interview was 53 min (see Table 1).

### Qualitative Data Analysis

Interviewers conducted a total of 73 semi-structured qualitative interviews, each lasting an average of approximately 50 min and audio recorded. Interviews were conducted in the local Bangla dialect, and later transcribed by the research assistants and translated into English (Ahmed et al., 2019b). A grounded theory approach was used in the analysis of the qualitative data (Strauss and Glaser, 1967), as well as thematic analysis, which is “a flexible and useful research tool that can potentially provide a rich and detailed, yet complex, account of the data” (Braun and Clarke, 2006: 78). Text from the transcripts was extracted to determine vulnerable populations’ perceptions about climate change, their experiences with the impacts of EWEs, and their coping strategies. The author also performed a paraphrased translation of the key information (Haq and Ahmed, 2017; Haq, 2018), a selection of which is included below. The thematic analysis procedures were conducted manually using the step-by-step guide suggested by Braun and Clarke (2006), as Kabir et al. (2016) did previously in examining perceptions about climate change impacts among coastal Bangladeshis. In the analysis some themes were found to be repeated by many respondents; in these cases, information one or two quotes included for each theme from among the many interviews (Saunders et al., 2018). The study used a comparative reporting method (Yin, 2003) to consider the different perspectives of respondents living in different types of EWE-prone areas.

## Results

### Understandings of Climate Change

This study collected information on how people living in areas with different vulnerabilities understand what climate change is and sought to determine whether this understanding varies depending on the type of vulnerability (flooding, cyclones, or drought). The results of the in-depth interviews show that there are similarities and differences in respondents’ understanding of climate change. Climate change was broadly perceived as manifesting as floods, heavy or excessive rainfall, high temperatures, thunderstorms, windstorms, droughts, and cyclones.

Many of those living in flood-prone areas mentioned irregular flooding and excessive rainfall among their perceptions about climate change. Some women defined it in terms of seasonal changes and irregular flooding; others included sunshine, cold, and heat in their definition. For example, one female respondent referred to it as excessive rainfall, flooding, and storms; another associated it with wind, heat, and rain. Another said:

“Climate change is defined as earthquakes, droughts, and floods.”

Male respondents held similar views on climate change; some also emphasized severity, mentioning very cold temperatures or fog in winter, very hot temperatures in summer, and heavy and erratic rainfall. One male respondent said:

“Climate change means sunshine and cold.”

In the cyclone-prone area, some highlighted rainfall, with little rainfall associated with drought. Some female interviewees defined climate change in terms of temperature, warming, and high temperatures. For example, one mentioned that climate change means that sometimes there is rain and sometimes there is drought, and that in the past there was less flooding than there is now; another noted that in the past the temperature in their area was relatively low, but now it is very high; and several mentioned an increase in precipitation and the feeling that it is warmer now than in the past. One said:

“Precipitation is decreasing day by day and it is low compared to the past.”

Another mentioned:

“Climate change means uncertainty, like sudden rainfalls, storms, cyclones, and sometimes extreme heat.”

In addition, both female and male respondents felt that climate change in their area would lead to more difficult days in the coming years. Male respondents also understood climate change as being about temperature, mostly mentioning the intensity and degree of temperature and emphasizing high and very high temperatures. Their comments were mostly based on a comparison of rainfall and temperatures between previous years and the present.

The results of the qualitative interviews show that people in the areas exposed to floods and cyclones have more similar opinions than they do with those in drought-prone areas. Interviewees in drought-prone areas focused on the occurrence of droughts and high temperatures when defining climate change. For example, one female interviewee mentioned that climate change is related to rainfall that sometimes occurs, the sun that is sometimes visible, warming, and high temperatures. Other respondents also focused on high temperatures, heavy rainfall, and heat waves. Some female respondents said that the summer feels warmer compared to the past; others focused on excessive rainfall, high temperatures, and cold winters. One said:

“The temperature is high in summer and does not feel cold in winter.”

In terms of defining climate change, respondents in the three regions gave their opinions based on experiences they usually encounter where they live. In addition, male respondents defined climate change in slightly more detail than their female counterparts and included more terms in their definition. Male respondents mentioned some of the terms used by female respondents, but were also very concerned about the intensity and severity of changes and comparisons with the past. They mostly used terms, such as high temperature, excessive rainfall, and very hot. This focus on weather conditions may be related to the fact that men are usually out of the house and work outdoors, for example in agriculture and fishing.

### Ideas on Why Climate Change and EWEs Occur

The survey also asked respondents about why they think climate change and EWEs occur. Respondents from vulnerable areas considered different questions when giving their explanations, and their perceptions were haped by both their experiences and their beliefs. These explanations revolved around factors such as the will of God or the Almighty, the reduction in river depth, water coming from the mountains, and gradual deforestation.

In flood-prone areas, some female respondents mentioned that flooding is due to water coming down from the hills and flooding the lowlands, and others that it is due to heavy rains and God’s will. Others noted that the depth of the river was decreasing. Some male respondents also mentioned that water comes from the mountainous areas to flood the lowlands, or that climate change and floods were caused by God punishing people for unacceptable behavior. One male interviewee mentioned:

“People do bad things, and God Almighty makes it worse by ordering EWEs with severe consequences. If everyone followed their religion, God Almighty would not wreak these disasters. If someone does something that is forbidden in their religion, God Almighty sends down heavy rain, causing a flood.”

In cyclone-prone areas, many male and female respondents also cited the decline of trees as a cause of climate change and flooding. Male respondents cited religious beliefs and the decline of rivers as causes, while one female respondent cited vehicle emissions. One female respondent said:

“Before, the temperature was not so high because we had more trees in our area. The trees helped make our area cooler. Now you can see that there are very few trees here. Now many vehicles in our area also create exhaust, which affects the heat. In the past, there were fewer vehicles here.”

Another woman interviewed said:

“In our area there were different kinds of trees and crops. Now there is only shrimp farming.”

Male respondents said that they see few trees in their area, that the environment is not in good shape now, and that they suspect the weather conditions will not change for the better in the future. While the women tended to focus on what causes climate change, the men also tended to worry about the future and express concern that it will become more difficult to live on the planet. This was encapsulated by one male respondent:

“We have a lot of flooding here. We have very few, if any, trees in this area. The rainfall is decreasing at the same time as the trees are decreasing. If we want to improve the situation, we need to plant more trees. The situation will continue to deteriorate. Mutual understanding between the locals in our area is decreasing. I think in a few years it will be difficult to stay here.”

Another said:

“There is no reason for the change in climate and the occurrence of EWEs. They are due to the change in the flow of the river. If we stop the normal flow of rivers, there will be storms. Today, some rivers are declining.”

In the drought-prone areas, some female respondents doubted whether climate change is in fact occurring and believe the situation has not changed. They demonstrated confusion about what exactly is causing extreme temperatures and extreme rainfall. As one mentioned:

“In my experience, I do not see climate change, and the scenarios for the occurrence of floods are the same in our area.”

Male respondents, on the other hand, placed full store in religious beliefs in explaining climate change and the occurrence of EWEs. One male respondent said:

“The Almighty gives us excessive heat and excessive rain. Climate change and floods are about a decision of the Almighty. The Almighty decides what is for the good of human beings.”

### Impacts of Climate-Change-Related Extreme Events

We asked the interviewees in the flood-prone areas to describe the challenges they face during flooding. Various problems were mentioned, including destruction of crops, inability to work, lack of shelter, hunger, lack of cooking facilities, having to borrow money with interest, waterborne diseases, children not being able to go to school for a long period, inadequate sanitation, lack of drinking water, loss of money to put into farms, no money to repair or rebuild damaged houses, problems sleeping, foul-smelling water, fear of snake bites, and fear of children drowning.

The women interviewed were concerned about limited housing, lack of food availability, hunger, and disruption of children’s education. One female respondent said:

“We had nothing to eat and we were hungry. We could not cook food because we had no water. We did not have a place to live during the floods because our house was under water.”

Another said:

“The hailstorm damaged our fields. Children can’t go to school during floods.”

Very important issues raised by respondents included hygiene problems, health problems, and loss of money needed to run their farms. One woman respondent said the following:

“Our fields were under water. My husband lost the money he had invested in the farm.”

Another said:

“Because of the water, sanitation is a big problem for women. We cannot afford a healthy, sanitary latrine.”

And another said:

“People’s blood pressure was increasing. Many people died of heart attacks and some died of pneumonia. Children are facing many illnesses.”

Another female respondent mentioned lack of clean water, fear of children drowning, lack of space in homes, and fear of snakebites:

“I have seen houses drowned and destroyed by flooding. Young children drowned and died. The floodwater had an unpleasant smell and was undrinkable. During the big floods, some families took refuge in local schools. However, there was not enough space for all the people. So we had to stay in our homes, and it was difficult.”

Another woman said:

“There was a lot of water in our house. During that time, a snake bit me. I still remember that experience.”

Male respondents reported their hardship and the credit trap they fall into in the face of frequent flood events. They could not find a way to recover from the damage and fell prey to opportunists who lend money at high interest rates to poorer people, and the poor get caught in a spiral of borrowing. One male respondent said the following:

“Our house and crops were damaged by the flood and hailstorm. The roof of our house was totally damaged. We could not live in our house. I lost so much, like money, crops, and the house, and I was in debt. A hailstorm damaged my house and I had to repair it. Again, a flood also damaged my house. I had to repair my house again. I spent a lot of money and it was a financial loss. I could no longer go to work or earn money. As a result, I had to borrow money afraid I couldn’t pay back the loan because of the high interest.”

Another said:

“Our transportation was hindered. The water overflowed and got into our houses. We could not buy food without a boat, and our financial situation was not good enough to buy one. We have been starving day and night, along with our child. Since we are day laborers, we have to buy food every day. We are also afraid of snakes at night. We cannot sleep. We cannot eat rice every day.”

And another said:

“During floods, we can’t ensure our children’s education and we are not able to send our children to school.”

And another said:

“I can’t work during a flood, and if I don’t work for two days, it is difficult for me to provide for my family. I cannot rebuild my house because I have financial problems and the fish are often taken by the floods. We can’t protect the fish and we have financial challenges.”

For respondents from the cyclone-prone area, their interviews reveal a different set of challenges. The main difficulties they are vulnerable to include finding shelter during a cyclone, interruption of crop production due to salt water getting in their fields, disease, lack of drinking water, damage to trees, livestock, and houses, lack of food, problems with sewage disposal, death of young children, and the high price of commodities during a crisis.

Many of the women interviewed said that they could not find a place to bathe because their homes were destroyed by the cyclone, and that sanitation was poor in the shelters where they had to take refuge. One woman stated:

“I lived through cyclones Aila and Sidr. Our house and trees were destroyed during Sidr and Aila. I could not find a safe place where the storm and rain would not do damage. In our area, the water is salty and we cannot grow crops with this salt water. We often have trouble finding drinking water because of the salt water. We had to buy drinking water. We also had problems bathing regularly.”

Another said:

“I saw Aila and Sidr. We could not save our house, our belongings, or our trees. We could only save our lives. We went to the cyclone center and left everything behind. We stayed there for about three years. When we returned to our village, we found nothing. During Aila, many people died. We suffered from diarrhea, cholera, etc. With the salt water we could not produce anything. During the events, we women had many problems like lack of food and clothing, no housing, and no sanitary facilities.”

And another stated:

“Many people died during Aila, especially those who were out fishing. Many children cannot swim. Many also died falling from destroyed houses. Many mothers could not save their young children because there wasn’t time. My uncle died during Aila. We could not find their bodies. We could not access money, clothes, etc. At the time, many NGOs came to help us, but they were insufficient.”

And another said:

“Before, we could buy one kilo of rice for 15 to 20 taka and we could easily feed our family if we bought two or three kilos of rice per day. But now, a kilo of average-quality rice costs 35 to 40 taka. After Aila, the price got high.”

Male respondents made similar points, and raised another very important issue, namely, when a woman is pregnant, it is very difficult to get her to a safe place. There are risks to the unborn child and there are difficulties in obtaining medical care if health problems arise during childbirth. They also mentioned that their livelihoods depend on fishing, but as they can no longer fish they have difficulty providing food for their family, especially their children. A few mentioned that there is help or financial support from the government or non-governmental organizations, but it is difficult for them to access it unless they have personal relations with officials or the ability to negotiate with them. Those who have a connection with a politically influential person can more easily obtain financial support. Some of the respondents also said that they need to go to another area to find work and earn money to feed their family and overcome the hardships caused by the cyclones. One male respondent stated:

“All my crops were completely destroyed by Cyclone Aila. Fishing was not possible. I could not find work and had no income for a year after Cyclone Aila. Many women were pregnant at that time and had to find a safe place. We asked some of our neighbors to host us at the cyclone shelter. Some of them replied that they hadn’t found a place for us yet but they could take my wife.”

Another said:

“We had two fields where we were growing rice. I lost all my crops. We also lost our house. My neighbor lost his son. Many people received 20,000 taka from the local government, but I could not get this money. Those who did had a connection in the local government. Many people got the money twice through their own connections. Since I did not have such a connection, I could not get it. Aila affected almost all of us and nobody could lend us money, so I had to borrow money from four or five people. After Aila, we lost our source of income. Here income opportunities are limited. So I had to go somewhere else where I could earn money to feed my family.

Respondents from drought-prone areas had different points to make about the impact of EWEs. They referred to the disruption of crop cultivation, increased use of fertilizers affecting the taste of food, decreased availability of fish, high prices for daily commodities, scarcity of drinking water due to the lower water table in a drought, diseases linked high temperatures, deaths in heat waves, and the need for additional money for irrigation.

Some women mentioned difficulties in getting around in an extreme drought, increased use of fertilizers affecting the taste of food, disruptions in crop production due to high temperatures, poor crop yields, and unavailability of potable water. One said:

“People can no longer go outside because of the high temperatures. We used to grow many types of crops, but now it is difficult to grow crops. The increasing use of various fertilizers recently is changing the taste of our food.”

Another woman said:

“We don’t get fish from the river anymore, as there are fewer now than before. Daily goods are quite expensive now. Due to the drought, we cannot produce sufficient crops. People who are highly dependent on agriculture are suffering even more because they mostly can’t turn a profit.”

And another said:

“In our area, it rains very little, but there are many storms. The temperatures are high in summer. The tube wells do not work properly. It is very difficult to pump water from the tube wells because the water level drops due to the high temperatures. The high temperature brings on many illnesses such as fever or cough in our children.”

Male respondents, for their part, emphasized difficulties in feeding their family and meeting daily expenses when crops fail due to drought and lack of sources of water for growing crops. They mentioned that they have to buy water from owners of deep wells at high prices, which are controlled by a syndicate. Trouble arises when these high prices are paid without then achieving expected crop yields. This forces people to move from farming into some other kind of work. One male respondent said:

“Due to high temperatures and drought, we farmers face water shortages. In the summer, the water level drops. People have to get water from other sources, but they are very expensive.”

Another said:

“We feel the extreme heat all the time. I think the temperature will rise in the next few days. We had our own land and my father was able to feed our large family only through farming. Now we can’t grow anything on this land. The yield has decreased so much that people can’t feed their families. Sometimes I can’t get enough water for the harvest. And so my crops are damaged. People who have enough money can get water from a deep tube well, but those who are poor can’t afford it. In our area, it is difficult to make a profit from farming. Because of the bad weather, many people have started to change their occupation and do other work instead.”

### Coping Strategies Against Climate Change and EWEs

Interviewees were asked how they were adapting to and coping with the negative effects of the crisis. In the flood-prone area, many respondents said they had to borrow money to support their families, rebuild their houses, buy medicine for their children, and work their fields, but at high interest rates. Some also mentioned that relatives that had stopped eating in order to save money to repay their loans, others that they built beds out of bamboo and bricks and cooked using a tin can.

Some female respondents mentioned how they organized their cooking during the flood period. Many women also reported the burden of loans and the difficulties this causes for their families, as each missed installment increases the interest to be paid. One female interviewee said:

“No, we didn’t get help from anyone. We had to borrow money to survive. Later, we had to work hard to pay back the loan.”

Another said:

“We borrowed some money and put it into the farm, but the whole crop collapsed in the water. We are worried about how to repay the money. My mother-in-law and father-in-law stopped eating because of the need to repay. We made a stove out of a tin can. We also made a bed out of bamboo and bricks and slept at an elevated level. We went to the market by boat and did some shopping. After the flood, we started working hard to try to pay back the loan.”

And another said:

“We had financial problems and sought help from other people to solve it. I borrowed money from a lender and bought groceries. I used some of the money to farm my land. I needed some money when my child was born at that time. I also borrowed money from a lender to get my child treated.”

Male respondents focused on having had to borrow money and difficulties in repaying the loan. They also mentioned doing extra work and fishing to help repay their loans. One said:

“I’ve been fishing and trying to do some extra work. I’ve borrowed money from lenders with interest. Those who are financially strong borrow money with interest. Sometimes I couldn’t make my payments on time and as a result had to pay extra interest.”

Another said:

“A few people died in the flood. We made a raft out of banana trees to save ourselves. My wife borrowed money. When the flood came, we built a makeshift bed out of bamboo and stayed on it. Some people stayed at school and some decided to stay in their homes at night. Some went to their relatives’ houses.”

In the cyclone-prone area, interview participants mentioned similar problems. They reported that even though some NGOs had helped them during the crisis, both men and women had to take work as day laborers to maintain their families, such as tilling for the men and housecleaning for the women. One interesting point was one female interviewee mentioning wanting to start a business and businesspeople being respected, but not having the funds to do so—pointing to women having the courage to become entrepreneurs. One female interviewee mentioned.

“I saw Cyclone Aila. Many people went to the cyclone center closest to us. They provided food, housing materials, work, and so on. After Ira, we didn’t have jobs. The recycling center gave us different jobs, such as day labor: men were involved in tilling the soil and women in cleaning.”

Another said:

“People respect those who are in business. I would like to have a business, but I don’t have the capital. I can’t start a business. Now only rich people can engage in fishing. We poor people can’t do it anymore.”

And another said:

“In those days, many NGOs, such as Caritas or others, helped us prevail and rebuild our houses. Some NGOs gave us money without expecting us to repay it. Some government organizations also came to help us by providing food, clothes, and even cash.”

One female respondent said:

“Women need safety and security when they go out. For example, when there is a cyclone, men can take boats to rescue people in danger and repair riverbanks. Men can collect relief goods as they are distributed. There are jobs for day laborers on other people’s land. But these jobs are far from our villages. Men can go there to find work. My husband goes to other areas to harvest rice.”

For their part, male respondents mentioned that they raise cows and goats to sell to support their families. They also catch fish to sell, and they need to borrow money. Some mentioned that relief is offered by different organizations, but sometimes they need to pay to access it, even though it is supposed to be freely distributed. One male interviewee said:

“Now I earn money by raising cattle and sheep on other people’s land. In this way, I can sustain my family for 15 to 20 days a month. But it’s difficult, and I have to borrow to maintain my family. We received a lot of help from NGOs as well as from foreigners. They gave us daily necessities such as rice, pulses, biscuits, water, and so on. I go out fishing, and if I can catch some fish, I can sell it and get some money. I borrow money. If I catch fish and make money, then I can repay some of the debt.”

Another said:

“The government as well as some NGOs provided us with enough relief, but we don’t get that relief directly from them. We get it from our union president or members, and sometimes, we need to pay to get it. If our president or members provided us with proper relief, then we would be much better off in times of crisis. However, they do not distribute the government relief in a proper way.”

And another said:

“I usually borrow money from NGOs that are not in my area. Many NGOs come to help us. They provide us with enough dry food. But even though the NGOs come to provide us with relief, we don’t receive it. Many people get that relief from our local members or chairmen by giving them money. And so we cannot benefit from the help offered by NGOs or the government.”

In the drought-prone areas, interviewees had similar comments. In all three areas, many people mentioned that they borrowed money to cope after an EWE. Some female interviewees mentioned that they work as seamstresses, work they can do from home, as women are not culturally allowed to spend long periods away from home. One said:

“We borrow money and then use it to buy everyday items, medicine, and so on. I don’t have to pay any interest on those loans. There are several people who give me money.”

Another said:

“Everyone may not like women working. Some people may not like it. Sometimes my neighbors say I should not do hard work because it could affect my health. I take their advice positively because they like me. I think people like me to do sewing, because this work does not require going out. I can stay at home and do this work.”

Another said:

“We borrowed money from a local NGO and we need to repay it in monthly installments. We need another four years to repay it all.”

## Discussion

This study examines how people living in three kinds of areas vulnerable to EWEs—due to flooding, cyclones, and droughts—understand climate change, how they experience its effects, the strategies they use to cope with it, and whether these vary according to the type of EWE. The results of the in-depth interviews show that there are similarities and differences between regions in all these respects. Although the study did not set out to focus on the gender perspective, from the qualitative data it emerged that there are gender differences in these respects.

People in the three vulnerable areas understand climate change and EWEs based on their own experiences of them, perceiving that climate change manifests as floods, heavy or excessive rainfall, storms, high temperatures, high winds, droughts, and cyclones. Many of those living in flood-prone areas mentioned irregular flooding and excessive rainfall. Women tended to understand climate change in terms of seasonal changes, cold, and heat, while men tended to emphasize the severity of climate change, mentioning that climate change means very cold or very hot summers, heavy and irregular rainfall, and fog in winter. In cyclone-prone areas, women understood climate change in terms of warming or high temperatures, while both men and women felt that it would lead to more difficult days in their area in the coming years. In drought-prone areas, many people focused on the occurrence of drought and high temperatures in their understanding of climate change. Poortiga et al. (2019) also found such variation in a cross-country comparison on perceptions about climate change in Europe.

In their understanding of climate change, respondents in all three regions based their views the experiences they typically encounter where they live (Salick et al., 2009; Turner et al., 2009; Ayeb-Karlsson et al., 2016). The male respondents explained their definition of climate change in slightly more detail and included more terms than did the female respondents, and tended to focus on the intensity and severity of changes in comparison with the past.

Regarding what respondents thought are the causes of climate change and EWEs, their experiences and beliefs with EWEs shaped their perceptions (Bunce et al., 2010; Speranza et al., 2010). Factors mentioned included the hand of God, lowering river levels, water coming down from the mountains, and the gradual loss of trees. In flood-prone areas, women mentioned flooding, the lowlands, God’s will, and the river getting more shallow (Alam et al., 2017), while men mentioned an increase in sin and climate change and EWEs as God’s punishment. In cyclone-prone areas, both women and men cited tree felling as a cause of climate change and flooding. In drought-prone areas, responses showed that women were not sure about climate change and perceive the situation as unchanged, and did not know what might be the cause of extreme temperatures and rainfall, while men’s religious beliefs were the main basis for explaining climate change and EWEs (Debela et al., 2015; Abegunde, 2017; Haq and Ahmed, 2017).

In the flood-affected areas, women worried about limited housing options, food scarcity (Ayeb-Karlsson et al., 2016), interruption of their children’s education, hygiene problems, loss of money for reinvestment into their farms, lack of clean water, fear of their children drowning, lack of space in their houses, and fear of snakebites; for their part, men reported the credit trap they fall into to be able to provide food for their families in the face of shortage, and felt that the only way to recover from the damage is to succumb to opportunistic money lenders who lend to poorer people, who then get caught in a debt spiral.

In the cyclone-prone areas, women respondents reported not being able to find anywhere to bathe after losing their houses in a cyclone and that the makeshift shelter had sanitation issues (Kabir et al., 2016; Mallick et al., 2017). The men mentioned difficulties in getting their pregnant wives to safety and in getting medical help should problems arise during childbirth. They also mentioned that as they could no longer fish, they had lost their livelihood and found it difficult to feed their families. They also mentioned the difficulty of accessing help or financial support from the government or NGOs for those who do not have personal connections government officials.

In drought-prone areas, people cited disruptions to farming, increased use of fertilizers and their effects on the taste of food, reduced availability of fish, high grocery prices, scarcity of with drinking water due to dropping groundwater levels, and the need for extra money for irrigation. Women mentioned difficulties in getting around in very hot weather, the increased use of fertilizers affecting the taste of food, and poor crop yields due to high temperatures. Men worried about how they could provide for their families’ daily needs when crops fail and about the consequences of having to buy water for irrigation at high prices from owners of deep wells only to achieve poor crop yields anyway, compelling them to stop farming and seek other work (Toufique and Islam, 2014).

In terms of coping strategies, many respondents from the flood-affected areas reported that they had to borrow money to support their families, rebuild their homes, buy medicine for their children, and run their farms, but at high interest rates. Both men and women reported the burden of loans, difficulties in repaying, and the increased interest with each missed payment (Wisner et al., 2004), with men having to work extra and go out to fish to supplement the family’s income. In the cyclone-affected area, people reported that despite NGO support during the crisis, men and women had to take work as day laborers to be able to meet their families’ needs. Some men raised cows and goats to sell and had to borrow money, and there were complaints about having to pay local gatekeepers to be able to access relief that was supposed to be freely distributed. Respondents from the drought-prone areas expressed similar views to those from flood- and cyclone-prone areas (Uddin et al., 2020), with many having had to take out loans to deal with the effects of drought or women taking on sewing work they could do from home, in keeping with cultural norms (Ahmed et al., 2019b).

In short, experiences with the impacts of EWEs and socio-demographic and cultural aspects influence people’s perceptions about climate change and the coping strategies they adopt in response to EWEs (Fahad and Wang, 2018; Evertsen and van der Geest, 2020), and vulnerable individuals routinely innovate in their coping strategies (Haq, 2018; Smith and Mayer, 2018; Sultana et al., 2020).

## Conclusion

People living in vulnerable areas perceive climate change based on their own experiences of it (changes in precipitation and temperature), and the importance of climate change to them varies depending on the type of EWE in question. While there are differences between people living in areas vulnerable to different types of EWE in how they understand climate change, there is little gender difference in this regard. People’s experiences of EWEs are an important factor in understanding their negative impacts on their regular activities and their livelihoods, of which there are many: crop loss, lack of shelter and limited safe spaces for women, lack of earning capacity, inability to afford to rebuild their lost home, food insecurity, and risk of young children and the elderly dying. People affected by EWEs often need to accrue debt as a coping strategy in order to feed their families and meet their daily needs, rebuild their homes, and run their farms, and if they miss an installment, the next one increases. It is clear that the climate is set to change rapidly and that EWEs will be a frequent and regular occurrence and a primary determinant of different aspects of human life. People will have to experience and cope with the impacts and challenges posed by these events as a matter of course. The regularity of EWEs coupled with the regularity of borrowing in response to their effects will lead to a “double whammy”: on one hand the immediate effects of climate change and EWEs, and on the other a spiral of debt.

Issues of poverty, food insecurity, access to education, inequality, and health are worsening and will be severely affected by changes in climate conditions and EWEs. Delaying the development of mitigation strategies to address the adverse situations faced by vulnerable populations and avoiding addressing the problems as a holistic issue integrating different stakeholders and accounting for its socioeconomic, demographic, and cultural dimensions will hinder the achievement of the UN’s Sustainable Development Goals in developing countries like Bangladesh. This paper therefore proposes detailed examination of the debt spiral in order to provide policymakers with insights to help them update their coping and adaptation strategies to help vulnerable populations avoid falling into this trap in inevitable future crises.

## Data Availability Statement

The datasets presented in this article are not readily available because the qualitative data were collected by ensuring the respondents that it will be confidential and will not be shared. Then, they provided their consent and collected the relevant data. Requests to access the datasets should be directed to shahatiq1@yahoo.com.

## Ethics Statement

Ethical review and approval was not required for the study on human participants in accordance with the local legislation and institutional requirements. The participants provided their written informed consent to participate in the study.

## Author Contributions

SA: conceptualization, data collection and analysis, and drafting, revising, and finalizing of the manuscript.

## Conflict of Interest

The author declares that the research was conducted in the absence of any commercial or financial relationships that could be construed as a potential conflict of interest.

## Publisher’s Note

All claims expressed in this article are solely those of the authors and do not necessarily represent those of their affiliated organizations, or those of the publisher, the editors and the reviewers. Any product that may be evaluated in this article, or claim that may be made by its manufacturer, is not guaranteed or endorsed by the publisher.
